# Change in the optic nerve sheath diameter after deflation of a pneumatic tourniquet: a prospective observational study

**DOI:** 10.1038/s41598-021-04457-4

**Published:** 2022-01-11

**Authors:** Ha-Jung Kim, Yeon Ju Kim, Jiyoung Kim, Hyungtae Kim, Young-Jin Ro, Won Uk Koh

**Affiliations:** Department of Anesthesiology and Pain Medicine, Asan Medical Center, University of Ulsan College of Medicine, 88, Olympic-ro 43-gil, Songpa-gu, Seoul, 05505 South Korea

**Keywords:** Medical research, Signs and symptoms

## Abstract

Applying a pneumatic tourniquet provides surgeons with a bloodless surgical field. However, application of the tourniquet induces various physiological changes. We evaluated the effect of tourniquet deflation on the intracranial pressure by using ultrasonography to measure the optic nerve sheath diameter (ONSD) in patients undergoing lower limb surgery. The ONSD was measured in 20 patients at five time points: after anesthetic induction (T0) and immediately before (T1), immediately after (T2), 5 min after (T3), and 10 min after tourniquet deflation (T4). Hemodynamic and respiratory variables were recorded. The ONSD showed significant differences at each point (P < 0.001). The ONSDs at T2 and T3 were significantly greater than that at T1 (P = 0.0007 and < 0.0001, respectively). The change in the end-tidal carbon dioxide partial pressure (EtCO2) was similar to the change in the ONSD. The change in the ONSD was significantly correlated with the change in the EtCO2 after tourniquet deflation (r = 0.484, P = 0.030). In conclusion, the ONSD, as an indicator of intracranial pressure, increased after tourniquet deflation in patients undergoing lower limb surgery. This was correlated with an increased EtCO2 and arterial carbon dioxide partial pressure.

**Trial registration:** ClinicalTrials.gov (NCT03782077).

## Introduction

Although there has been a debate on the use of pneumatic tourniquet in orthopedic surgery, they do provide surgeons with a bloodless surgical field, which improves the surgical environment. In addition, pneumatic tourniquets reduce the surgical time and intraoperative blood loss^1,2^; therefore, they are still widely used during lower limb surgery. However, inflation and deflation of the tourniquet induces physiological changes, including alterations in the pH, arterial oxygen partial pressure, arterial carbon dioxide partial pressure (PaCO_2_), potassium ion levels, and lactate levels^3^. In particular, tourniquet deflation causes carbon dioxide (CO_2_) that has accumulated during the ischemic period to be released into the systemic circulation. The resultant elevation in PaCO_2_ is followed by an increase in the intracranial pressure (ICP)^4^. Although this increase in cerebral blood flow (CBF) has not much clinical significance and it is compensated and tolerated in most healthy patients, the increase in cerebral blood volume may cause an increase in ICP in patients with decreased intracranial compliance^5–7^. Increased intracranial pressure can induce deleterious outcomes, including cerebral ischemia^7,8^. Therefore, cautious monitoring and thorough management of the ICP is required in patients undergoing lower limb surgery, who have or are at high risk of cerebrovascular disease, using a pneumatic tourniquet.

Many studies have shown that ultrasonography of the optic nerve sheath diameter (ONSD) has good diagnostic accuracy to detect intracranial hypertension^9^. Increased ICP is transmitted to the subarachnoid space surrounding the optic nerve, causing optic nerve sheath expansion^10^. Therefore, increased ICP could be estimated based on the ONSD, which can be measured in a non-invasive manner that could be applied easily in orthopedic patients.

In the present study, we aimed to investigate alterations in the ICP using ultrasonography to measure the ONSD after deflation of the pneumatic tourniquet in patients undergoing lower limb surgery. We also evaluated the factors related to changes in the ONSD.

## Results

In November and December 2018, 21 consecutive patients scheduled for undergoing knee surgery, using a pneumatic tourniquet were screened for eligibility. Among them, one refused to participate in the study, and 20 were enrolled, all of whom finished the study without drop-out. Ultimately, 20 patients were included in the final analysis. The patients’ baseline characteristics and intraoperative data are shown in Table 1.

**Table 1:** Demographic data.

	(n = 20)
Age (years)	67.1 ± 6.9 (63.8–70.3)
Height (cm)	154.3 ± 6.4 (151.3–157.3)
Weight (kg)	61.8 ± 8.2 (58.0–65.7)
Sex (male/female)	1/19
Alcohol consumption (heavy/social/none)	1/0/19
Smoking (smoker/ex-/none)	1/1/18
Operation time (min)	160.3 ± 34.9 (144.0–176.6)
Anesthetic time (min)	130.5 ± 37.0 (113.2–147.8)
Tourniquet time (min)	98.4 ± 27.2 (85.6–111.1)
Amount of fluid (ml)	547.5 ± 222.7 (450.0–645.0)

Data are expressed as mean ± standard deviation (95% confidence interval) or number of patients.

**Figure 1:**
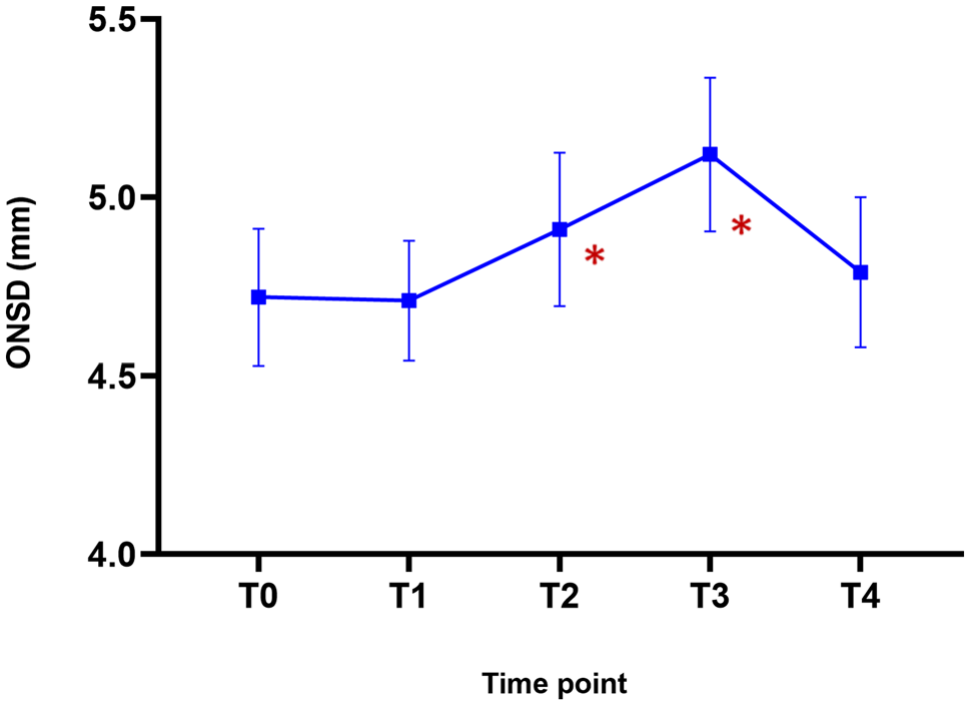
Comparison of optic nerve sheath diameters (ONSDs) measured at after anesthetic induction (T0) and immediately before (T1), immediately after (T2), 5 min after (T3), and 10 min after deflation (T4). The ONSDs were significantly increased at T2 and T3 as compared with T1. The dot and error bars indicate the mean and standard deviation, respectively.

**Figure 2:**
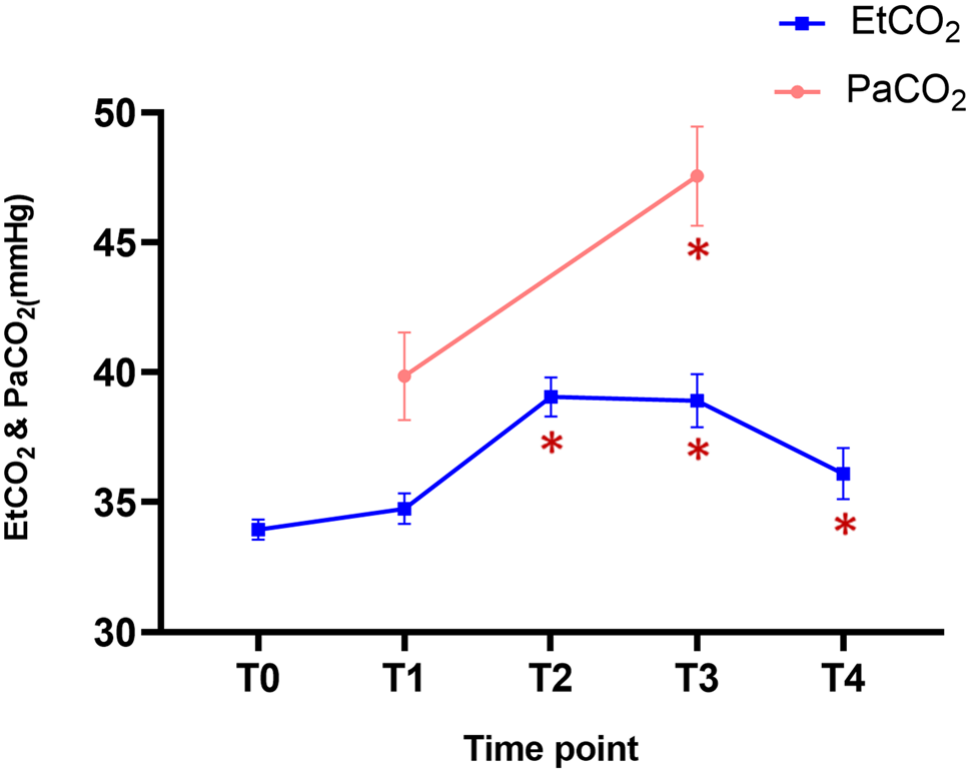
The blue lines indicate that compared of EtCO_2_ measured at after anesthetic induction (T0) and immediately before (T1), immediately after (T2), 5 min after (T3), and 10 min after deflation (T4). EtCO_2_ were moderate correlated with ONSD. The red line indicates that compared of PaCO_2_ measured at immediately before (T1) and at 5 min after deflation (T3).

The ONSD showed significant differences at each time point (P < 0.001; Table 2 and Fig. 1). A post hoc analysis demonstrated that ONSDs at T2 and T3 were significantly greater than that at T1 (P = 0.0007 and < 0.0001, respectively). However, the ONSD at T4 was no different from that at T1. The change in the end-tidal carbon dioxide partial pressure (EtCO_2_) was similar to the change in the ONSD. The EtCO_2_ levels at T2, T3, and T4 were significantly higher than that at T1 (P < 0.0001, P < 0.0001, and P = 0.001, respectively). The PaCO_2_, obtained through arterial blood gas analysis, was also significantly higher at T3 than at T1 (P < 0.0001; Fig. 2). Other intraoperative variables at each time point, including hemodynamic and respiratory variables, are shown in Table 3. Mean arterial pressure (MAP) significantly decreased over time, and the MAPs at T2, T3, and T4 were lower than that at T1. Plateau airway pressure (PAP) and peak inspiratory airway pressure (PIAP) were significantly lower after tourniquet deflation compared with the PAP and PIAP at T1 except for PAP at T3. The body temperature was lower than before the tourniquet deflation only at T3 and T4.

**Table 2:** Intraoperative optic nerve sheath diameters.

Time points	Optic nerve sheath diameter	P*-*value
T0	4.72 ± 0.41( 4.53–4.91)	–
T1	4.71 ± 0.36 (4.54–4.88)	
T2	4.91 ± 0.46 (4.69–5.12)	0.0007*
T3	5.12 ± 0.46 (4.90–5.33)	< 0.0001*
T4	4.79 ± 0.45 (4.58–5.00)	0.2164

Linear mixed model with multiple comparison using Scheffe’s method were used. Data are presented as mean ± standard deviation (95% confidence interval).

*T0* after anesthetic induction, *T1* immediately before tourniquet deflation, *T2* immediately after tourniquet deflation, *T3* 5 min after tourniquet deflation, *T4* 10 min after tourniquet deflation.

*P < 0.05 compared with T1.

**Table 3:** Intraoperative hemodynamic and respiratory variables.

	T0	T1	T2	T3	T4
EtCO_2_ (mmHg)	33.95 ± 0.82	34.75 ± 1.25	39.05 ± 1.60*	38.90 ± 2.17*	36.1 ± 2.10*
PaCO_2_ (mmHg)	NA	39.85 ± 3.60	NA	47.55 ± 4.08*	NA
MAP (mmHg)	73.50 ± 10.09	85.43 ± 13.17	70.60 ± 9.64*	67.83 ± 8.04*	64.63 ± 6.97*
BT (℃)	35.96 ± 0.41	36.28 ± 0.52	36.22 ± 0.56	35.77 ± 0.52*	35.63 ± 0.51*
PAP (mmHg)	14.45 ± 2.85	15.35 ± 3.16	14.65 ± 3.04*	14.90 ± 3.14	14.80 ± 3.08*
PIAP (mmHg)	13.90 ± 2.82	14.95 ± 3.13	14.10 ± 2.95*	14.30 ± 3.04*	14.20 ± 3.07*

Linear mixed model with multiple comparison using Scheffe’s method were used. Data are presented as mean ± standard deviation (95% confidence interval).

*EtCO*_*2*_ end-tidal carbon dioxide partial pressure, *PaCO*_*2*_ partial pressure of arterial CO_2_, *MAP* mean arterial pressure, *BT* body temperature, *PAP* plateau airway pressure, *PIAP* peak inspiratory airway pressure, *T0* after anesthetic induction, *T1* immediately before tourniquet deflation, *T2* immediately after tourniquet deflation, *T3* 5 min after tourniquet deflation, *T4* 10 min after tourniquet deflation.

*P < 0.05 compared with T1.

The change in the ONSD was significantly correlated with the change in the EtCO_2_ from immediately before tourniquet deflation to 5 min after tourniquet deflation (Pearson’s correlation coefficient [r] = 0.484; P = 0.030; Table 4). However, there was no significant correlation between the change in the ONSD and the change in the MAP.

**Table 4:** Pearson correlation coefficient between the change of the ONSD and EtCO_2_ and MAP.

	T2		T3		T4	
	Pearson’s *r*	P-value	Pearson’s *r*	P-value	Pearson’s *r*	P-value
EtCO_2_	− 0.044	0.853	0.484	0.030*	0.111	0.641
MAP	− 0.345	0.135	− 0.343	0.138	− 0.277	0.235

Pearson’s correlation coefficients(*r*) with the level of significance (*P* ≤ 0.05). *EtCO*_*2*_, end-tidal carbon dioxide partial pressure; *MAP*, mean arterial pressure; *T2*, immediately after tourniquet deflation; *T3*, 5 min after tourniquet deflation; *T4*, 10 min after tourniquet deflation. *P < 0.05 compared with T1.

## Discussion

In this prospective observational study, we found that the ONSD, as an indicator of the ICP, increased after tourniquet deflation in patients undergoing lower limb surgery. This was correlated with an increased carbon dioxide after tourniquet deflation.

A few methods can be used to monitor the ICP in real-time: the intraventricular catheter and the intraparenchymal catheter^11^. The intraventricular and intraparenchymal catheters are considered the gold standard techniques based on their accuracy^12^. However, these invasive techniques carry the highest risk of complications and are not always feasible^12^. Therefore, noninvasive methods for indirectly estimating ICP are attracting the attention of researchers: transcranial Doppler and the ONSD. Transcranial Doppler and the ONSD are both non-invasive, low-cost, and accessible at the bedside. However, transcranial Doppler has several disadvantages. For example, it is difficult to find an adequate acoustic window in some populations and the technique only measures arterial blood flow and volume, without any information on other factors that contribute to the ICP^13,14^. Conversely, the ONSD is easily visualized in most cases. Moreover, a pressure rise in the intracranial compartment leads to a shift of CSF from the intracranial subarachnoid space into the optic subarachnoid space^15^, and an increase in ONSD is an early manifestation of increased ICP. Thus, the ONSD is useful for monitoring the ICP during surgery.

During tourniquet inflation, the oxygen supply decreases because there is no arterial or venous blood flow in the limb; anaerobic processes then occur in the ischemic area^3^. Deflation of the tourniquet allows reperfusion of the ischemic limb, which can cause increases in oxygen consumption and CO_2_ production 2 min after tourniquet release^16,17^. The current study corroborated previous investigations, in which the EtCO_2_ significantly increased immediately after tourniquet deflation and the increased levels were maintained for up to 10 min. In addition, the PaCO_2_ level was significantly higher after tourniquet deflation. This rapid increase in the PaCO_2_ after tourniquet deflation is associated with transient increases in the CBF^18,19^. Ellingsen et al. showed that the CBF response started less than 30 s after the PaCO_2_ began to increase, and that it required up to 2 min to reach a peak value^20^. Changes in the ICP are rapidly transmitted to the optic nerve sheath, with no apparent temporal lag^21^. In the present study, the EtCO_2_ showed a peak value immediately after deflation, but the ONSD, which is an indicator of the ICP, showed the highest value after 5 min of deflation. However, this inconsistency with other studies may have arisen because the two variables were measured in different ways in previous studies—in the present study, all variables were measured intermittently, including the EtCO_2_ and ONSD; therefore, we could not identify the exact peak time of each variable.

The PaCO_2_ was not the only factor affecting the ICP and ONSD. Blood pressure may also influence the ICP by altering the CBF. After tourniquet deflation, blood pressure decreases because vasodilatory substances are released into the circulation^22^. In the present study, blood pressure decreased immediately after tourniquet deflation and was maintained for 10 min. Previous studies have demonstrated that a blood pressure drop is associated with decreased CBF^23,24^. However, we found that the EtCO_2_ showed moderate correlation, while the MAP was not apparently correlated with the CBF. Based on this result, we speculated that the PaCO_2_ was a more important determinant of the ICP than blood pressure. In addition, the MAP after tourniquet deflation stayed between 60 and 150 mmHg in the present study. CBF autoregulation typically occurs between these values^25^.

In healthy individuals, the respiratory drive is stimulated towards compensation and increases breathing when hypercapnia occurs^26^. If hyperventilation is not adequately achieved after tourniquet deflation, the CBF velocity increases^27^. The transient increase in CBF may have a detrimental effect in patients at risk of cerebrovascular accident, including brain injury^7^. When patients are self-breathing, changes to the ICP caused by hypercapnia after tourniquet deflation can be compensated by increased breathing. In previous studies on the tourniquet time and ICP^28^, recovery of spontaneous breathing after tourniquet deflation may have influenced results, because the researchers did not continuously infuse any neuromuscular blocking agents. In the present study, we aimed to assess the effect of tourniquet deflation itself; therefore, we continuously infused a neuromuscular blocking agent under a train of four monitoring. The present study demonstrated that tourniquet deflation was followed by related ONSD dilation and increases in the PaCO_2_ and EtCO_2_ levels without any compensation by self-breathing. To assess whether compensation via spontaneous breathing affected changes in the ICP and ONSD, future studies should compare the ONSD after tourniquet deflation between controlled ventilation and spontaneous breathing groups.

The present study had the following limitations. First, it was conducted under general anesthesia using sevoflurane. Inhalational anesthetics, such as sevoflurane have a cerebral vasodilatory effect and can increase the CBF and ICP in a dose-dependent manner^29^. However, sevoflurane was maintained at approximately 1 minimal alveolar concentration; therefore, its effect was likely minimal^30^. In addition, sevoflurane has not been shown to affect the autoregulation of cerebral vasculature, unlike other inhalational anesthetics^31^. Second, the ONSD and PaCO_2_ were not measured continuously, thus we could not identify the exact peak time of increase in the ONSD and PaCO_2_ after tourniquet deflation. The ONSD and PaCO_2_ cannot be monitored continuously. However, we did show that ONSD increases within a few minutes of tourniquet deflation and then decreases towards the baseline level within 10 min. Third, it is difficult to draw a definite conclusion due to our study was an observational study and the number of enrolled patients was small. Nevertheless, we cautiously believe that ONSD measurements could improve outcomes by detecting ICP increases after tourniquet deflation in patients at higher risk of cerebrovascular accident. In conclusion, tourniquet deflation caused an increase in the ONSD in patients undergoing lower limb surgery, and this increase was associated with an increase in the PaCO_2_. Furthermore, sonographic ONSD monitoring might provide useful information regarding changes in the ICP that occur during lower limb surgery using a tourniquet.

## Methods

### Study design

This prospective observational study was performed at Asan Medical Center, University of Ulsan College of Medicine, Seoul, Korea. The study protocol was approved by the Institutional Review Board of Asan Medical Center (protocol number: 2018-1172, approval date: 01/SEP/18), and written informed consent was obtained from all patients. The study was conducted according to the 1964 Helsinki declaration and its later amendments. We registered the study on ClinicalTrials.gov (NCT03782077).

### Patients

The study included patients who met the following criteria: (1) the American Society of Anesthesiologists physical status I–IV^32^, (2) age between 18 and 80 years, and (3) knee surgery with pneumatic tourniquet scheduled at our center. We excluded patients with cerebrovascular, neurological, or ophthalmic diseases, as well as those who refused to participate or had any contraindication to general anesthesia or tourniquet use.

### Anesthetic management

The recruited patients entered the operating room without any premedication. Standard monitoring was performed, including peripheral oxygen saturation, electrocardiogram, non-invasive blood pressure, EtCO_2_, and body temperature. General anesthesia was induced using 2 mg/kg of propofol and 50 µg of fentanyl. When the eyelash reflex was lost, 0.6–0.8 mg/kg of rocuronium was intravenously injected, and the lung was mask-ventilated using 5–6 vol% sevoflurane in 80% oxygen for 3 min. When the patients reached an adequate anesthetic depth, an appropriately sized supraglottic airway device was inserted. The esophageal temperature probe was also inserted via a supraglottic airway device to measure body temperature. Anesthesia was maintained using 1.5–2 vol% of sevoflurane in a 50% oxygen/nitrous oxide mixture at a rate of 2L/min. The concentration of sevoflurane was controlled according to the bispectral index level and minimal alveolar concentration of inhalation agent to avoid overdose sevoflurane. Mechanical ventilation was adjusted to maintain the EtCO_2_ level at 35 mmHg with a tidal volume of 6–8 mL/kg and a respiratory rate of 10–12/min. To eliminate the effect of self-respiration compensating hypercapnia, rocuronium was continuously infused to maintain a train of four count of 1–2 throughout the surgery. After anesthetic induction, the radial artery was cannulated for invasive arterial blood pressure monitoring and blood gas analysis. Crystalloid solution including plasmalyte was administered at a maintenance dose to maintain normovolemia. To control pain, we administered an ultrasound-guided femoral nerve and sciatic nerve block by injecting 40 cm^3^ of 0.3% ropivacaine. A pneumatic thigh tourniquet was applied as close as possible to the limb root, and it was inflated to a pressure of 300 mmHg. All surgical procedures were performed with the tourniquet inflated; after the surgery, the tourniquet was deflated. When the ONSD measurements were completed, the inhalation agent and rocuronium were discontinued, and the neuromuscular blockade was reversed using sugammadex. After recovery from general anesthesia, patients were transferred to the postanesthetic care unit.

### Measurements

**Figure 3:**
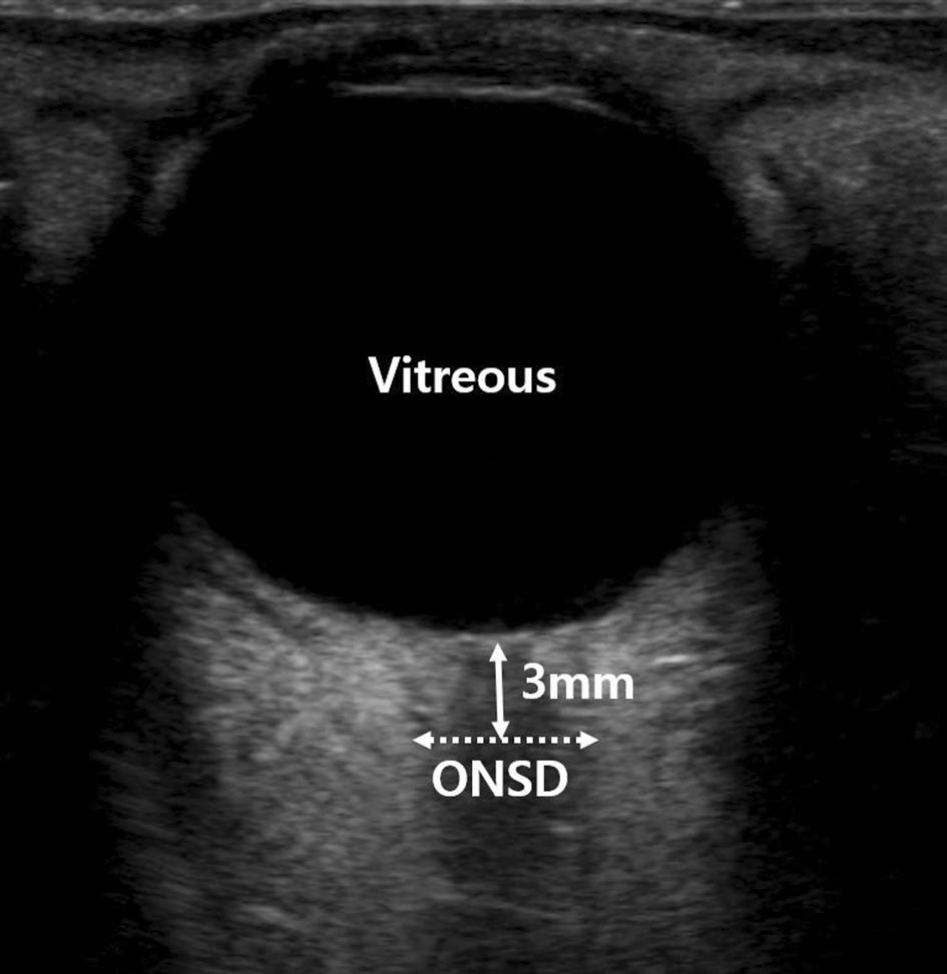
Measurement of optic nerve sheath diameter (ONSD) by ultrasonography. The outer diameter of the optic nerve sheath was measured 3 mm behind the optic disc.

The primary outcome of this study was a change in the ONSD, measured by ultrasonography after tourniquet deflation. Ultrasound imaging was performed by two investigators trained in ocular sonography using the 6–13 Hz linear probe of a single ultrasound machine (S-Nerve; SonoSite, Bothell, WA, USA). Patients were placed in the supine position with their heads in the neutral position. The probe was located on the closed upper eyelid in the transverse plane and moved slightly to identify the optimal image between the vertical hypoechoic band and the retrobulbar echogenic fat tissue. The outer diameter of the optic nerve sheath was measured 3 mm behind the optic disc (Fig. 3). We measured the ONSD twice in each eyeball, and used the average values of the four measurements in the analysis^33^. The measurements were performed at the following five points: after anesthetic induction (T0), immediately before the tourniquet deflation (T1), immediately after the tourniquet deflation (T2), 5 min after tourniquet deflation (T3), and 10 min after tourniquet deflation (T4).

MAP, respiratory variables, and body temperature were measured at the same time points. Respiratory variables included the PAP, PIAP, and EtCO_2_ and they were measured by the ventilator machine (Drager Primus, Lubeck, Germany). Arterial blood gas analysis was also performed to ensure accurate PaCO_2_ measurement at T1 and T3. In addition, the patients’ baseline characteristics and tourniquet time were recorded.

### Statistical analysis

Based on our clinical experiences, the mean ONSD after anesthesia induction was 5.0 ± 0.5 mm. When an ONSD change of 10% was considered significant, the calculated sample size with a power of 0.9 and an alpha-error of 0.1 was 18 patients. Considering dropout, we decided to enroll 20 patients.

The data we obtained were recorded in a standardized case report form. In the analysis of repeated measured data, including ONSD, a linear mixed model was used. Furthermore, we performed multiple comparison using Scheffe’s method to compare variables between two time points. Pearson’s correlation coefficient analysis was used to analyze how the ONSD change was correlated with the EtCO_2_ change and MAP change. P-values of < 0.05 were considered statistically significant.
